# Placental Complications

**Published:** 1861-10

**Authors:** 


### Placental Complications.

*Treatment of Placental Presentations.*—The practice of removing the placenta, in cases of placental presentation, seems to have been well known and practiced in Manchester for years, and originated in the practice of the late Mr. Kinder Wood. Mr. Wood says:]

If we find so much exhaustion as to make us fear the effects of further hemorrhage, during artificial delivery, the first step after passing the hand must be to detach the whole of the placenta; by this the hemorrrhage will be completely suppressed, for the effect of passing the hand through the os uteri, and throwing off the placenta, will always be to produce so much contraction as to arrest the bleeding from the small decidual or uterine mouths. It is satisfactory to know that the child is rarely living in these cases of exhaustion, its blood being poured out through branches of the placental structure, along with that of the mother; and when brought down, its appearance, like that of the mother, is bleached and exsanguined. The time required to separate the placenta is very short, and the loss of blood during the attempt exceedingly trifling. I know from experience, that when the placenta is wholly detached, the hemorrhage will cease.

In some cases I have been called to attend, when the ordinary method of delivery has been adopted, the patient died, and this led me to modify the practic, by detaching the placenta, rupturing the membranes, and then delivering the child; but after due consideration I was again induced to vary my plan, and in those cases where we can have no hope in saving the patient if we proceed to delivery, however well the operation be conducted. I have no hesitation in recommending that the placenta be separated completely, and the membranes ruptured, and that the hand be withdrawn immediately upon this being effected, leaving the child and placenta behind. By this practice the patient will be placed precisely in the situation which occurs in the most favorable cases of recovery by the natural efforts. I conceiive no fact in midwifery rests upon a more solid foundation, than that this hemorrhage will cease upon separating the placenta, and by this practice the patient is placed in as favorable a situation as is possible for recovery. Time will be gained to support her by proper means, and which can be used with greater freedom, as the hemorrhage is infallibly suppressed by this operation. *Part* xi., *p*. 195.

*Placental Presentation—Extraction of the Placenta before the Child in Cases of.*—All obstetric authors agree that no complication in midwifery occasions more anxiety to the practitioner, or danger to the patient, than hemorrhage from placental presentation, and the results of practice fully support this opinion. Of 174 cases which Dr. Churchill has collected, 48, or nearly one-third of the mothers, died. Dr. Simpson has attempted a still more extensive analysis, and finds that of 399 cases recorded, in 115 the result was fatal to the mother, a mortality as great as that of malignant cholera in 1832–33, and twice as great as the average in cases of lithotomy. Looking at the results, any attempt to diminish such fearful mortality is entitled to the most careful notice of the profession. Hitherto two great principles of treatment, which some have supposed to be applicable to the two different stages of the complication, have been pursued, viz: 1st, the evacuation of the liquor amnii; and 2d, delivery of the child by turning.

[Some accoucheurs, as Drs. Burns and Hamilton, Baudelocque, Capuron, and others, think this the *only* mode of treatment advisable under any circumstances in these cases. Drs. Merriman, Conquest, Dewes and Denman, are unanimous in the opinion that turning must be resorted to.]

Cases of placenta prævia, says Dr. S., frequently occur in practice, in which neither of the two preceding plans can be successfully adopted—where the artificial evacuation of the liquor amnii is insufficient to moderate the hemorrhage to a safe degree, and where forced delivery by turning is inapplicable or extremely dangerous if adopted. In these and other cases, I would beg to submit to my obstetric brethren an additional principle of treatment, viz: The complete separation, and, if necessary, extraction of the placenta before the child. Obstetric pathologists seem unanimous in the opinion that all the more formidable varieties of hemorrhage, which occur from the uterus in the latter months of utero-gestation, or the earlier periods of labor, are attributable to the separation of the vascular connections between the placenta and the interior of the uterus, and the escape of blood from the vessels which are laid open in consequence of this separation. Paradoxical as it may appear, there are sufficient grounds and facts for believing, that when the placenta is separated slightly and partially, the chance of fatal hemorrhage to the mother is greater than when the disunion of the organ is more entire and complete. Various authors have detailed cases in which the death of the mother speedily took place, though the portion of the placenta separated from the uterus was exceedingly small.

Dr. S. has collected notes of 141 cases in which the placenta was expelled before the child, which he throws into a tabular form, and divides into—1st, Cases in which a considerable interval elapsed between these two occurrences; 2d, Cases in which the interval was less than ten minutes; 3d, Cases in which the child was born immediately after the placenta, or along with it; and 4th, Cases in which the period is not mentioned. I believe, says Dr. S., that the data which I have collected are amply sufficient to establish, as a great physiological and practical fact, that, when the placenta, in cases of unavoidable hemorrhage, is once completely detached from its connections with the interior of the uterus, the accompanying flooding in general entirely ceases, or become quite moderate and inconsiderable in quantity.

*Summary of Results.*—1. The total separation and expulsion of the placenta before the child, in cases of unavoidable hemorrhage, is not so rare an occurrence as accouchers appear generally to believe. 2. It is not by any means so serious and dangerous a complication as might *a priori* be supposed. 3. In nineteen out of twenty cases in which it has happened, the attendant hemorrhage has either been at once altogether arrested, or it has become so much diminished as not to be afterward alarming. 4. The presence or absence of flooding after the complete separation of the placenta, does not seem in any degree to be regulated by the duration of time intervening between the detachment of the placenta and the birth of the child. 5. In ten out of one hundred and forty-one cases, or in one out of fourteen, the mother died after the complete expulsion or extraction of the placenta before the child. 6. In seven or eight out of these ten casualties, the death of the mother seemed to have no connection with the complete detachment of the placenta, or with results arising directly from it, and if we do admit the three remaining cases (which are doubtful), as leading by this complication to a fatal termination, they would still only constitute a mortality, from this complication, of three in one hundred and forty-one, or about one in forty-seven cases. 7. On the other hand, under the present established rules of practice, one hundred and thirty-four mothers died in three hundred and ninety-nine placental presentations, or about one in three. *Part* xi., *p.* 203.

*Placenta Prævia—Partial.*—Rupture the membranes as soon as possible, and excite uterine action by friction, the application of a binder, ergot of rye, or galvanism.

*Complete.*—If the os is undilated and rigid, *plug* with a sponge dipped in vinegar and water; when the os is sufficiently dilated, seize a foot and deliver cautiously. In head presentation with a dead child, use craniotomy; and in both this case and that of foot presentation, rupture the membranes early through the placenta, by a silver catheter.

When the vital powers are much depressed, and the placenta partially detached, plugging is dangerous. So are the administration of secale cornutum, and delivery of the child, because they lower still more the powers of the sysfem. Detach completely the placenta, and employ galvanism, which both rouses the uterus, and stimulates generally.

Turn as soon as possible, extract the placenta, and get the uterus to contract: taking care to keep up the pulse. If the strength is too much exhausted for this, or if the os uteri will not admit the hand, separate the placenta if possible; rupture the membranes with the nail, and *plug the vagina completely*, then give ergot of rye, and apply cold, or galvanism. When the pulse rallies, withdraw the plug, and if possible turn; if this cannot be done, plug again and wait. It is plain that if we cannot deliver, we must directly stop the bleeding; and plugging in the case supposed, where the placenta is low down, is quite effectual.

Dr. Simpson advises when the hemorrhage and exhaustion are very great, and when turning, etc., are impracticable, on account of the undilated state of the os and cervix, especially in primiparæ, or the contracted state of the uterus itself, or the narrowness of the pelvis; or when turning is dangerous on account of the exhaustion of the mother, or not required for the safety of the child, on account of its death or prematurity, to *detach the placenta*, and leave the expulsion of the child to nature, if the hemorrhage, as is usually the case, ceases, and no particular complication arises. *Part* xv., *p.* 274–279.

*Retained Placenta.*—Empty the umbilical vein, and forcibly inject cold water, slightly acidulated with acetic acid; or if there is much hemorrhage, cold decoction of ergot, or solution of tannic acid. *Part* xv., *p.* 280.

*Placental Presentations.*—The practice recommended by Dr. Simpson, of entirely detaching the placenta, and leaving it, is likely to be of use in very many instances; as where there is such an unyielding condition of the soft parts as to prevent speedy delivery, or where the flooding has been going on for a considerable time, and the practitioner, on his arrival, finds the bleeding still continuing, and the woman so much exhausted that the sudden emptying of the womb would be dangerous. But this plan by no means supersedes delivery by turning, which, if adopted in a prompt and energetic manner, is a safe and proper method of treatment.

The practice of detaching the placenta is inapplicable in the most formidable cases—is as difficult to accomplish as turning, and does as much violence to the uterus—is almost certainly fatal to the child, and affords no increased chance of safety to the mother. If the cause of hemorrhage is ascertained to be placenta prævia, rupture the membranes, introduce the hand, and turn and deliver the child—provided that the os is sufficiently dilated, or easily dilatable; if it is not, wait, and act upon general principles till sufficient relaxation has taken place.

When copious and repeated hemorrhage occurs in the eighth or ninth month of pregnancy, and signs of labor do not appear, evacuate the liquor amnii, pass a conical plug into the os uteri, fill up the vagina behind it, and then give one or more doses of secale. The plug occasions the formation of a large coagulum within the uterus, the presence of which, combined with the irritation caused by the plug, excites uterine contraction. By the expulsion of the clot, the os is dilated, and we are then able to introduce the hand and turn with more facility. *Part* xvii., *pp.* 225–237.

*Placenta Prævia.*—The chief methods of treatment are: 1. The plug. 2. Puncturing the membranes. 3. Turning the child. Plugging is adapted to those cases which occur at the sixth or seventh month, when a continuous drain is going on after the first loss. The best method of plugging is to introduce strips of lint, pieces of sponge, or a silk handkerchief dipped in vinegar and water. Do not plug too tightly, or uterine contraction may be excited, and further separation take place. Puncturing membranes is adapted to those cases, not very severe, where the os uteri is only dilated to a slight extent, and turning is impracticable; it arrests the hemorrhage by lessening the vascular supply, and bringing down the presenting part of the child to act as a plug to the placental site. Turning is the grand remedy in placenta prævia; if performed at the proper time, it affords the greatest chance of safety to both mother and child.

The most prompt and efficient method in almost all cases is to introduce the hand, dilate the os uteri, separate the intermediate portion of placenta, rupture the membranes, seize one foot, turn, and deliver. In selecting the time when to do this, the great thing is to distinguish the dilatable character of the os uteri; if this be present, the extent of dilatation is of no importance comparatively. It requires great care that the dilating force be steadily applied and evenly distributed, so as to guard the cervix from laceration, which might prove fatal from hemorrhage. Version is sometimes prohibited by the immediate exhaustion of hemorrhage, or from rigidity of the os uteri, in spite of hemorrhage; in the former case plug and give stimulants freely, as a temporary resource. In the latter case, separate the entire connection of the placenta, rupture the membranes, and excite uterine action. *Part* xxxiv., *p.* 226.

*Encysted Placenta.*—That is, where the placenta is retained by the contraction of the os uteri after parturition, must be removed by gently dilating the os first by the placenta itself, if possible, or if this do not succeed, by the fingers, drawing gently at the cord, and at the same time having firm pressure made on the abdomen. *Part* xxxiv., *p.* 227.

*Placenta Prævia.*—In those terrible cases where the os is rigid and unyielding, and the flooding profuse, do not delay, but introduce one or two fingers through the os, and detach all that part of the placenta which adheres within the cervical zone, or region of dangerous placental seat. The contraction of the womb present in such a case as this, is the very element which will secure the success of this operation, as it constricts the mouths of the bleeding vessels. If contraction is not present, it must be induced by ergot of rye or galvanism. By the performance of this operation, moreover, the os and cervix are released from a mechanical impediment to dilatation. If necessary, the hemostatic process may also be assisted by the use of the plug. By these means the hemorrhage will have been arrested in the great majority of cases, but, if not, time will have been gained, and forced delivery rendered more easy.

*Part* xxxvi., *p.* 221.

*Placenta Prævia.*—A case is related in which, the os being dilated to the size of a small teacup, the separation (as advised by Dr. Barnes) of the whole circumference of the placenta which was presenting, sufficed at once to check the hemorrhage. By further separating the posterior portion of the placenta, the membranes were reached, and the presentation discovered to be a footling. The child was delivered in a short time alive, and the mother did well.

*Part* xxxix., *p.* 292.

*Placenta Prævia—Air Pessary.*—An interesting case is related by Mr. Jardine Murray, of Brighton, in which the os uteri being dilated sufficiently to admit two fingers, and syncope impending from hemorrhage, a flattened caoutchouc air-pessary was introduced between the wall of the uterus and the presenting surface of the placenta. The pessary being then inflated by means of the attached syringe, it acted admirably as a direct plug and dilator of the os uteri. Whenever, from time to time, the trickling of blood recurred, it was effectually checked by further dilatation of the pessary, by a few strokes of the syringe. In two hours the os was sufficiently dilated to admit the hand, and though turning was necessary, as the shoulder presented, the case was soon brought to a favorable conclusion. *Part* xl., *p.* 196.

